# Multiorgan failure following gastroenteritis: a case report

**DOI:** 10.1186/s13256-020-02402-z

**Published:** 2020-06-21

**Authors:** Joseph De Zylva, James Padley, Rassam Badbess, Maneesha Dedigama

**Affiliations:** 1 Medical Officer, South Coast District Hospital, 56 Bay Road, Victor Harbor, SA 5211 Australia; 2 Clinical Educator, Rural and Remote Health, College of Medicine and Public Health, Flinders University, Flinders Drive, Bedford Park, SA 5042 Australia; 3 Consultant General Physician, Flinders Medical Centre, Flinders Drive, Bedford Park, SA 5042 Australia

**Keywords:** *Clostridium perfringens*, *Enterococcus avium*, Sepsis, Gastroenteritis, Rural/regional hospital

## Abstract

**Background:**

This report highlights the first published case of fatal septic shock associated with *Clostridium perfringens* and *Enterococcus avium* bacteremia due to infective gastroenteritis.

**Case presentation:**

We report a case of hepatic infarction, abscess, and death following gastroenteritis in a 63-year-old Aboriginal man who initially presented to a rural hospital with suspected food poisoning. The patient had persistent fever and was commenced on empirical antibiotics. His blood culture results were positive for *Clostridium perfringens* and *Enterococcus avium*. He was transferred to a tertiary center but developed organ failure and refractory shock. Initial computed tomography of the abdomen was unremarkable, but repeat imaging showed small bowel enteritis, hepatic abscess, and infarction as a result of portal vein septic thromboembolism. Despite maximal intensive care treatment, including percutaneous drainage of hepatic abscess and broad antibiotic cover, the patient died 6 days after initial presentation.

**Conclusions:**

This case highlights the rare but commonly fatal course of sepsis associated with *Clostridium perfringens* bacteremia and demonstrates detrimental effects of coinfection with *Enterococcus avium*, including potential for rapidly seeding abscess formation. Lessons for rural practice are highlighted, including the need for urgent and early referral for intensive care support, particularly for patients with complex comorbidities.

## Background

Both *Clostridium perfringens* and *Enterococcus avium* are implicated in food poisoning from poultry. Historical data suggest that *C. perfringens* is the second most common cause of food poisoning after *Staphylococcus aureus* [1]. *E. avium* accounts for only 2.4% of cases of *Enterococcus* bacteremia and has been associated with visceral abscess formation [2, 3].

Since 1990, only 50 cases of *C. perfringens* bacteremia have been described [4, 5]. Sepsis from *C. perfringens* can be rapidly fatal, often because of massive hemolysis, with a reported mortality of 74% [4]. Few effective treatment options exist, although a survival benefit may be conferred by early antibiotic treatment, surgical source control, and/or hyperbaric oxygen therapy [4]. *C. perfringens* sepsis is often unrecognized at initial clinical presentation; however, the source of infection is usually related to underlying pathology involving the uterus, colon, or biliary tract [4, 5]. Although foodborne *C. perfringens* bacteremia has been described, fatal cases are exceedingly rare [6]. *E. avium* sepsis is poorly understood, owing to its relative infrequency of occurrence. We report a unique case of fatal complications associated with *C. perfringens* and *E. avium* septicemia following an initial foodborne illness.

## Case presentation

A 63-year-old Aboriginal man was referred by a general practitioner to a rural hospital emergency department. The patient presented with 24 hours of epigastric pain, vomiting, watery diarrhea, generalized muscle aches, and fever. The preceding night, the patient had eaten a piece of chicken that he had cooked after it had been refrigerated for a few days. The patient was an ex-smoker, was moderately obese, and had long-standing type 2 diabetes being treated with insulin and complicated by diabetic nephropathy (baseline serum creatinine 300–320 μmol/L) and peripheral vascular disease, ischemic cardiomyopathy (New York Heart Association [NYHA] class II symptoms), hypertension, and depression. Over the preceding 2 years, he had been managed in the outpatient heart failure clinic with review every 6–8 weeks. His heart failure symptoms had been deemed to be stable at his last review 6 weeks before presentation. The patient’s regular medications were low-dose aspirin, bisoprolol, fluvoxamine, gliclazide MR, insulin glargine, fenofibrate, simvastatin, and inhaled fluticasone/salmeterol. The patient lived alone and was functionally independent with activities of daily living.

Upon presentation to hospital, his initial examination was remarkable only for fever. Blood biochemistry showed raised inflammatory markers and chronically elevated creatinine (Table 1). The patient was admitted to the general medical ward of a rural district hospital for supportive treatment with a presumptive diagnosis of infective gastroenteritis. Initial management included intravenous fluid rehydration and antiemetics. His fluid status was monitored, and his regular medications, including insulin and inhaled bronchodilators, were continued.

**Table 1:** Hematology and biochemistry results in a patient admitted with gastroenteritis

	2 months prior	Day 1: 1745 h	Day 3: 1350 h	Day 3: 2030 h	Day 4: 1715 h	Day 5: 0510 h
Hb, g/L	136	136	118	119	88	83
Leukocytes, × 10^9^/L	7.09	10.55	11.48	9.44	14.02	7.61
Platelets, × 10^9^/L	299	180	112	90	72	59
Neutrophils, × 10^9^/L	4.91	10.05	10.1	8.5	12.9	6.9
Creatinine, μmol/L	303 (baseline 300–320)	327	460	577	377	322
Urea, mmol/L	13.4	16.6	26	28.3	18.3	14.3
Anion gap, mmol/L	20	23	29	32	26	26
Albumin, g/L	35	33	24	21	30	25
Bilirubin, μmol/L	4	66	414	380	355	345
ALP, U/L	91	112	100	108	95	104
ALT, U/L	33	330	2948	2155	2540	2622
AST, U/L	27	352	3663	2266	3531	3712
GGT, U/L	54	216	189	164	101	90
LDH, U/L	213	464	2010	1123	1831	1863
INR	0.9		1.7	1.4	1.3	

*Abbreviations: ALP* Alkaline phosphatase, *ALT* Alanine aminotransferase, *AST* Aspartate aminotransferase, *GGT* γ-Glutamyl transferase, *Hb* Hemoglobin, *INR* International normalized ratio, *LDH* Lactate dehydrogenase

Day 1 indicates the evening of presentation to the rural emergency department. On day 3, the patient developed intravascular hemolysis, acute liver and renal injury and was transferred to a tertiary center. Baseline blood results from 2 months prior to admission are also shown

**Fig. 1:**
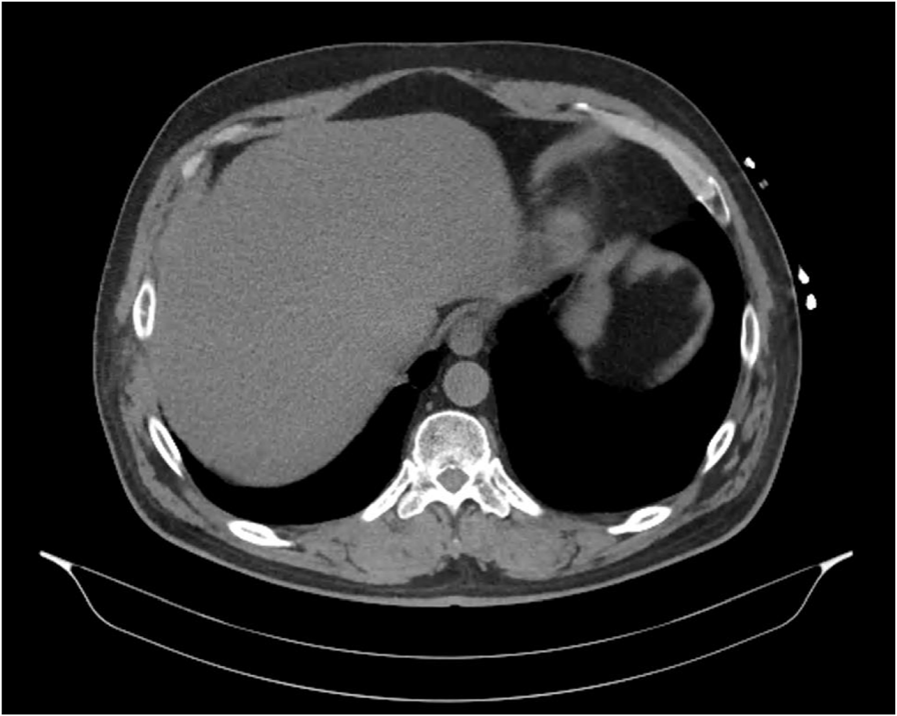
Computed tomography (CT) of the abdomen and pelvis in a patient with suspected gastroenteritis. Image was obtained within 24 hours of presentation to the hospital. CT demonstrated cholelithiasis but no other abnormality

Over the next 24 hours, the patient continued to have intermittent high fevers. Empirical antibiotics were commenced (intravenous piperacillin/tazobactam 4.5 g three times daily). Blood cultures subsequently grew *C. perfringens* and *E. avium*; both organisms were sensitive to penicillin. Computed tomography (CT) of the abdomen and pelvis demonstrated cholelithiasis but was otherwise normal (Fig. Fig. 1). Specialist infectious disease advice was sought on two occasions, which confirmed appropriate antibiotic management. The patient continued to remain clinically stable.

Viral nasopharyngeal swabs were also positive for human metapneumovirus. The results of fecal nucleic acid testing were negative for other common pathogenic organisms, including enteric viruses, parasites, and bacteria. The results of viral and autoimmune hepatitis screening were negative. Urine culture performed prior to initiating antibiotics was clear. Blood film obtained 48 hours after admission showed neutrophilic toxic changes.

On the third day after presentation, the patient became visibly jaundiced and complained of mild intermittent epigastric pain. Blood biochemistry showed acute renal and hepatic injury and hyperbilirubinemia suggestive of intravascular hemolysis (Table 1). The patient was urgently transferred to a tertiary center, given the risk of deterioration.

**Fig. 2:**
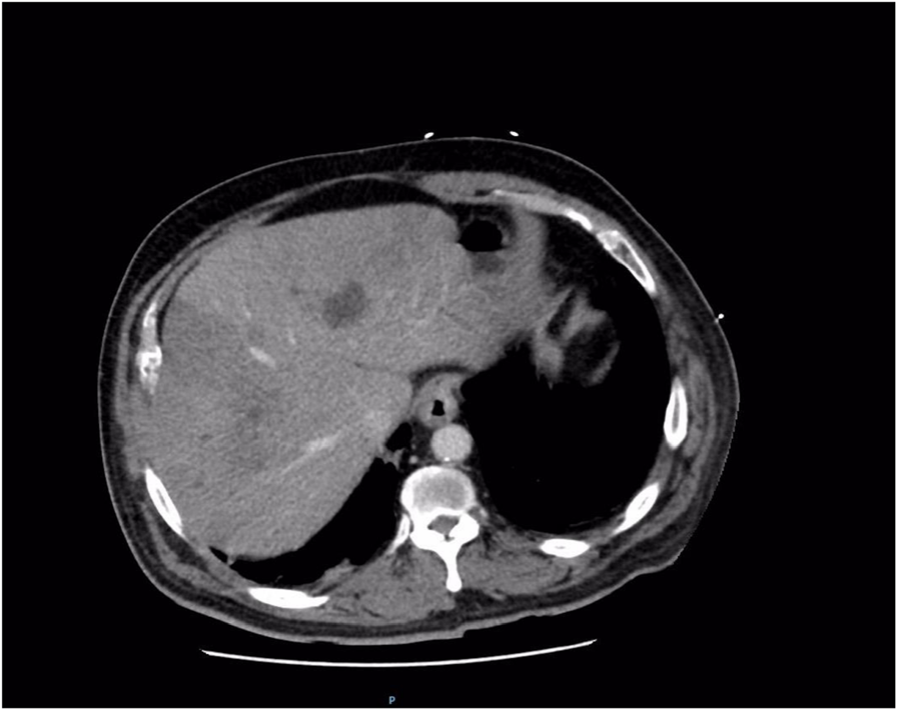
Computed tomography (CT) of the abdomen and pelvis 72 hours after presentation to the hospital showing multiple lobulated intrahepatic collections, infarction of hepatic segments 5 and 8, and septic occlusion of the portal vein. There were no gas locules associated with the abscesses. CT also demonstrated small bowel enteritis (not shown)

The patient’s condition significantly deteriorated over the next 24 hours, and he was admitted to intensive care with septic shock and renal failure. He required support with multiple inotropes and dialysis. Antibiotic cover was extended to include vancomycin, meropenem, and clindamycin (Table 2). Repeat CT of the abdomen and pelvis demonstrated small bowel enteritis, hepatic infarction, and portal vein thrombosis suspected to be septic thromboembolism. CT also demonstrated several hepatic hypodensities consistent with multiple abscesses, the largest measuring 41 mm in diameter (Fig. 2). Ultrasound-guided drainage was performed, and 35 ml of hemoturbid fluid was aspirated. The result of culture of the hepatic abscess was positive for *E. avium*.

**Table 2:** Summary of antibiotic management of a 63-year-old aboriginal patient with infective gastroenteritis complicated by sepsis and organ failure

	Day of admission				
	1	2	3	4	5
Antibiotic regimen		Piperacillin/tazobactam 4.5 g three times daily	Piperacillin/tazobactam 4.5 g three times daily	Piperacillin/tazobactam 2.25 g three times daily^a^	Piperacillin/tazobactam 2.25 g three times daily^a^
			Vancomycin 2.5-g loading dose then continuous infusion	Vancomycin continuous infusion	Vancomycin continuous infusion^b^
			Clindamycin 600 mg three times daily	Clindamycin 600 mg three times daily	Clindamycin 600 mg three times daily
				Meropenem 1 g three times daily	Meropenem 1 g three times daily^b^

On day 2, blood cultures from day 1 were positive for *Clostridium perfringens* and *Enterococcus avium* sensitive to penicillin. On day 3, the patient was transferred from a rural district hospital to a tertiary center, and his condition deteriorated over the next 24 hours

^a^Renally adjusted dose

^b^Discontinued on day 5 after specialist advice to de-escalate antibiotic regimen

The patient developed refractory septic and cardiogenic shock and required intubation and ventilation. Intubation was complicated by bronchial bleeding that required transfusion and endobronchial intervention. The patient had been temporarily commenced on therapeutic heparin for portal vein thrombosis; however, this was rapidly reversed with Prothrombinex®-VF (CSL Behring, Victoria, Australia) [7]. Transthoracic echocardiography demonstrated severe left ventricular (LV) dysfunction (estimated LV ejection fraction, 21%) but no evidence of valvular dysfunction or infective endocarditis. Despite maximal intensive care support, including intra-aortic balloon counter pulsation, the patient died 2 days after admission to intensive care, 6 days after his initial presentation.

## Discussion

The dramatic clinical course in our patient’s case demonstrates a rapid clinical deterioration associated with complications from *C. perfringens* and *E. avium* sepsis due to infective gastroenteritis. In our patient’s case, despite recognition of liver injury and hemolysis and early referral to a tertiary center, early antibiotic treatment and maximal intensive care support did not lead to recovery.

*C. perfringens* sepsis may present with nonspecific symptoms or with fever and abdominal pain and is associated with leukocytosis, anemia, thrombocytopenia, and liver dysfunction. *C. perfringens* bacteremia has been reported to arise from intra-abdominal (52.7%) or lower respiratory tract sources (19.4%) or to be associated with polymicrobial infection (54.8%) [8]. Previous case reports suggest that source control may lead to improved outcomes, although these cases were associated with a clear focus of infection [4, 5]. In our patient’s case, limited ultrasound-guided aspiration of a hepatic abscess was able to be performed; however, the disseminated sepsis and multiple small hepatic abscesses precluded other surgical intervention. Hyperbaric oxygen therapy was not indicated in the present case, although its use has been described in previous case reports in which there was a necrotizing focus of *C. perfringens* infection [4].

*E. avium* bacteremia is uncommon and accounts for only 2–3% of cases of *Enterococcus* bacteremia [9]. Little is understood regarding the clinical presentation of *E. avium* sepsis, given its infrequent occurrence, although it has a poor prognosis with an associated mortality rate of 24.5% [10]. *E. avium* disseminates predominantly from biliary (50.9%) or intra-abdominal sources (24.5%), and *Escherichia coli* is commonly detected in coinfection [10]. Risk factors for *Enterococcus* sepsis include biliary disease, diabetes, and solid cancers, particularly of hepatobiliary or gastrointestinal origin [9].

Both *C. perfringens* and *E. avium* are potentially pathogenic organisms with poor risk profiles when associated with systemic infection. This case highlights systemic effects of *C. perfringens* endotoxemia together with rapidly seeding abscess formation from *E. avium* bacteremia. In our patient’s case, early antibiotic management and limited source control did not prevent overwhelming sepsis and subsequent organ failure. The source of infection in this case could have been related to food preparation or precipitated by initial gastroenteric illness. It is not possible to distinguish the contribution of individual organisms to our patient’s initial clinical presentation and rapid deterioration, although this case highlights the detrimental effects of coinfection.

Our patient had risk factors and complex comorbidities that may have predisposed him to disseminated infection and subsequent organ failure. Diabetes, chronic kidney disease, and cardiac failure are independent risk factors of mortality from sepsis. The patient is likely to have had poor physiological reserve, which limited his ability to compensate for gastroenteric illness and endotoxemia. The risk of mortality from sepsis is increased by 2.27 times for NYHA class II heart failure and 2.36 times for estimated glomerular filtration rate (eGFR) less than 45 ml/minute compared with NYHA class I or eGFR > 60 ml/minute, respectively [11, 12]. Concurrent human metapneumovirus infection combined with endobronchial trauma may have further contributed to the patient’s poor outcome in this case.

## Conclusions/lessons for practice

- Suspect food poisoning in patients with multiple comorbidities

- Anticipate exaggerated physiological insults (e.g., associated with dehydration)

- Recognize complications associated with bacteremia; consider early referral and/or transfer

- *Clostridium perfringens* bacteremia:
  - High rate of mortality associated with complications, including hemolysis
  - Consider early transfer to a tertiary center hospital with intensive care capability
- *E. avium* bacteremia:
  - Rare cause of gastrointestinal illness; when present, consider polymicrobial infection and seeding abscess formation necessitating repeat imaging and drainage for source control

## Data Availability

Not applicable.
